# Perceptual Narrowing in Speech and Face Recognition: Evidence for Intra-individual Cross-Domain Relations

**DOI:** 10.3389/fpsyg.2018.01711

**Published:** 2018-09-12

**Authors:** Anna Krasotkina, Antonia Götz, Barbara Höhle, Gudrun Schwarzer

**Affiliations:** ^1^Department of Developmental Psychology, Faculty 06 – Psychology and Sports Science, Justus Liebig University Giessen, Giessen, Germany; ^2^Linguistics Department, University of Potsdam, Potsdam, Germany

**Keywords:** perceptual narrowing, perceptual reorganization, other-race effect, face perception, speech perception, habituation

## Abstract

During the first year of life, infants undergo perceptual narrowing in the domains of speech and face perception. This is typically characterized by improvements in infants’ abilities in discriminating among stimuli of familiar types, such as native speech tones and same-race faces. Simultaneously, infants begin to decline in their ability to discriminate among stimuli of types with which they have little experience, such as non-native tones and other-race faces. The similarity in time-frames during which perceptual narrowing seems to occur in the domains of speech and face perception has led some researchers to hypothesize that the perceptual narrowing in these domains could be driven by shared domain-general processes. To explore this hypothesis, we tested 53 Caucasian 9-month-old infants from monolingual German households on their ability to discriminate among non-native Cantonese speech tones, as well among same-race German faces and other-race Chinese faces. We tested the infants using an infant-controlled habituation-dishabituation paradigm, with infants’ preferences for looking at novel stimuli versus the habituated stimuli (dishabituation scores) acting as indicators of discrimination ability. As expected for their age, infants were able to discriminate between same-race faces, but not between other-race faces or non-native speech tones. Most interestingly, we found that infants’ dishabituation scores for the non-native speech tones and other-race faces showed significant positive correlations, while the dishabituation scores for non-native speech tones and same-race faces did not. These results therefore support the hypothesis that shared domain-general mechanisms may drive perceptual narrowing in the domains of speech and face perception.

## Introduction

The first year of an infant’s life is characterized by a fast attunement of perceptual mechanisms to the specific sensory inputs that infants encounter in their daily life. This process, known as perceptual narrowing, leads to a decline in the ability to discriminate or recognize stimuli that are not present or not relevant in the infant’s environment. So far, perceptual narrowing has been observed for visual as well as acoustic perception. In speech, very young infants can discriminate all kinds of speech sound contrasts but this sensitivity declines for many non-native sound contrasts within the first year of life (e.g., Werker and Tees, 1984; Höhle et al., 2009) and increases for native contrasts (e.g., Kuhl et al., 2006). Such perceptual narrowing may arise for vowels (around 6 months; e.g., Polka and Werker, 1994), consonants (around 10–12 months, e.g., Werker and Tees, 1984), and prosodic properties like lexical tone contrasts (between 6 and 9 months: e.g., Mattock and Burnham, 2006; Yeung et al., 2013; Götz et al., 2018) or word stress (Höhle et al., 2009).

Similar patterns have emerged in research on infants’ face perception. For instance, the face-sensitive N170 signal showed different properties for upright and inverted faces in adults, but similar properties for both orientations in infants (de Haan et al., 2002). Regarding the other-race effect, while Caucasian 3-month-olds discriminated between faces within four ethnic groups, Caucasian 6-month-olds discriminated faces within only two, and Caucasian 9-month-olds only discriminated Caucasian faces (Kelly et al., 2007). Similar results were also found for Chinese infants (Kelly et al., 2009). Experience with faces of other races seems to slow or modify perceptual fine-tuning toward faces of one’s own race (Heron-Delaney et al., 2011; Spangler et al., 2013). Thus, with increasing age, specific experience with face categories leads infants to fine-tune their face-processing system to those faces that are most relevant in their environment (Schwarzer, 2014).

The similarities between the perceptual narrowing processes in speech and face perception have led to the suggestion that these domains share some underlying developmental mechanisms (Maurer and Werker, 2014). However, research examining interactions between these domains in perceptual narrowing has started to appear only recently. For instance, Minar and Lewkowicz (2017) found that 10- to 12-month-old Caucasian infants could still discriminate Asian faces when the faces articulated the vowel /a/ but not when the articulating faces were presented silently or with a non-speech sound superimposed on the speech sound.

The present study investigated relations between perceptual narrowing in these two domains by testing the effects of perceptual narrowing in both domains in Caucasian monolingual infants: We tested 9-month-old German learning children on their ability to discriminate same-race and other-race faces, as well as non-native Cantonese tone contrasts in separate experiments using an infant-controlled habituation-dishabituation paradigm.

## Methods

### Participants

Fifty-three healthy, full-term Caucasian infants of German origin (*M* = 287, 92; range: 274–302 days, 24 girls and 29 boys) took part in our study. All infants were from monolingual German-speaking (without local dialects) households, with at least one parent in every household reporting some university-level education. Infants had no direct contact with persons of Asian descent according to a questionnaire administered to their parents. Thirteen additional infants were excluded from the final sample because of fussiness (*N* = 5), insufficient quality of eye-tracking calibration (*N* = 6), or technical problems during the experiment (*N* = 2). Infants were randomly assigned to either Group A or Group B. Group A participated in the face task in which other-race faces were used and in the non-native speech task (*N* = 27). Group B participated in the face task in which same-race faces were used and in the non-native speech task (*N* = 26). The order of the face and speech tasks was counterbalanced in both groups.

Our study was conducted in accordance with the German Psychological Society (DGPs) research ethics guidelines. The experimental procedures and informed consent protocols were approved by the Offices of Research Ethics at the Universities of Giessen and Potsdam. Written informed consent was obtained from all parents of the infant participants prior to their participation in experiments.

### Stimuli

#### Speech Stimuli

Non-native speech stimuli consisted of Cantonese CV syllables (/tɕ^h^i/) with mid-level (tone 33) or high-rising (tone 25) tone variants taken from the study by Yeung et al. (2013). For each tone, four distinct tokens were used. The duration of each individual tone was 40 ms. For each trial, each tone was presented repeatedly with 1 s intervals between repetitions at a volume of 75 dB for the duration of the trial. A previous study had shown perceptual narrowing for exactly these stimuli in German infants between 6 and 9 months (see Götz et al., 2018 for more details).

#### Face Stimuli

Face stimuli consisted of colored photographs of six other-race Asian (Chinese origin) and six same-race Caucasian (German origin) women, on a white background. All faces were presented in three poses: frontal, ¾ to left, and ¾ to right. On each photo the women looked straight at the camera with the hair, neck, and shoulders being visible. We edited the photos in Photoshop CS3 to make them matched in head size, and also made the skin-tone, eyes, head, and lip-color as similar as possible. Each photo was presented in the middle of the screen, appearing as 12.5 cm (10.98° visual angle) wide and 16.5 cm (14.47° visual angle) tall. Faces were paired within each ethnicity according to similarity ratings collected in a pilot experiment. A follow-up pilot experiment confirmed the occurrence of the ORE in 9-month-old infants using these face pairs.

### Procedure

Parents were informed about the general purpose of the study and the experimental procedure, but were blind to the hypotheses. Parents gave written consent for their child’s participation. During the experiment infants sat on their parent’s lap at a distance of approximately 65 cm from the 23.8″ display with a resolution of 1920 pixels × 1080 pixels, and an integrated Tobii tx300 eye-tracker with a sampling rate of 300 Hz. Parents were instructed to close their eyes and stay silent during the experiment. Each testing started with a 5-point infant calibration procedure. The calibration was repeated until it was successful for all five points for up to four maximum attempts. The data from infants who failed the calibration procedure were excluded from the final sample.

We used an infant-controlled habituation-dishabituation procedure for both the speech and face tasks. Within both habituation and test trials, stimuli were presented until infants looked away from the screen for 2 s, or until a maximum trial length of 40 s was reached. The average looking time during the first three habituation trials served as the baseline for the habituation criterion. The habituation phase continued in sets of three trials, until the average looking time for a set of three trials decreased to below 50% of the average from the first three trials. The habituation phase continued until either this habituation criterion was reached, or until a total of 18 habituation trials had been presented. Infants who failed to habituate (*n* = 7) were exclude from the final sample.

After habituation, infants proceeded to the test phase, where they were sequentially presented with the habituated stimulus and a novel stimulus of the same type, with each infant being randomly assigned to see the habituated stimulus either first or second. E-Prime version 2.0 (Psychology Software Tools, Pittsburgh, PA, United States) was used for stimuli presentation.

#### Speech Task

During the speech task infants were habituated to one of the two tones. A silent rotating animation of a colorful circle presented on the screen was used as an attention getter between trials. During test trials, the infants heard repetitions of the habituated tone, and the second (novel) tone in a sequential random order in order to eliminate order effects. A checkerboard pattern was presented on the screen whenever infants heard the tones during both habituation and test trials.

#### Face Task

During the face task, infants were habituated to photos of one person in three different poses, alternating in random order in sequences of three. To direct infants’ attention to the screen, a neutral audio signal was played as an attention getter before the start of each habituation trial. During the test trials, the previously habituated and a novel face of the corresponding condition were shown sequentially (in the frontal pose) in random order.

### Data Analysis

**FIGURE 1:**
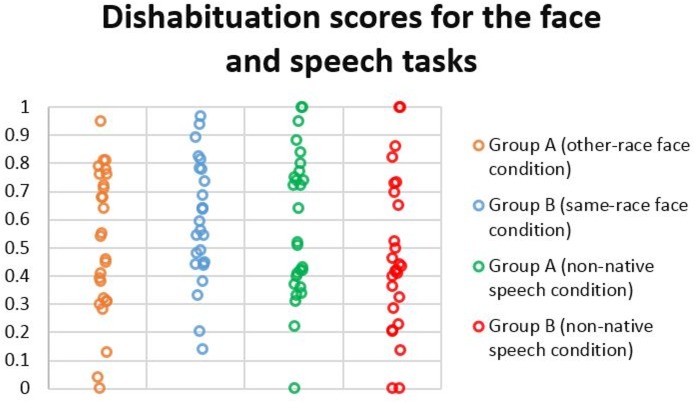
Dishabituation scores for the face and speech tasks.

Dishabituation scores for each infant in the speech and face tasks (**Figure 1**) were calculated by dividing the fixation time toward the novel stimulus by the sum of the fixation times toward the novel and habituated stimuli during test trials. Based on previous eye-tracking research with infants (Liu et al., 2011; Wheeler et al., 2011; Xiao et al., 2014) we defined fixations by a minimum duration of 100 ms within a 30 pixel radius. Tobii Pro Studio was used to analyze the eye-tracking data. The fixation times used for these calculations came from the area of interest covering the whole head in the case of the face task, and the entire checkerboard screen in the case of the sound task. Dishabituation scores above 0.5 indicated a preference for the novel stimulus, and dishabituation scores below 0.5 indicated a preference for the habituated stimulus. One sample *t*-tests were used to test the dishabituation scores against chance level.

Finally, to determine whether there was a relation between infants’ dishabituation scores in the speech and face condition, we calculated the Pearson correlations between the dishabituation scores in the speech and face tasks for each group of infants.

## Results

The *t*-tests in the face tasks revealed that only the infants in the same-race face condition (Group B) showed a significant dishabituation score (mean dishabituation score = 0.591, *SD* = 0.214*; t*_25_ = 2.181, *p* = 0.039; *d*_z_ = 0.43), while the infants in the other-race face condition (Group A) did not (mean dishabituation score = 0.517, *SD* = 0.255; *t*_26_ = 0.347, *p* = 0.732; *d*_z_ = 0.03). In the speech task, we did not find a significant dishabituation score in either Group A (mean dishabituation score = 0.577, *SD* = 0.259; *t*_26_ = 1.551, *p* = 0.133; *d*_z_ = 0.3), or Group B (mean dishabituation score = 0.529, *SD* = 0.276; *t*_25_ = 0.538, *p* = 0.596; *d*_z_ = 0.11).

**FIGURE 2:**
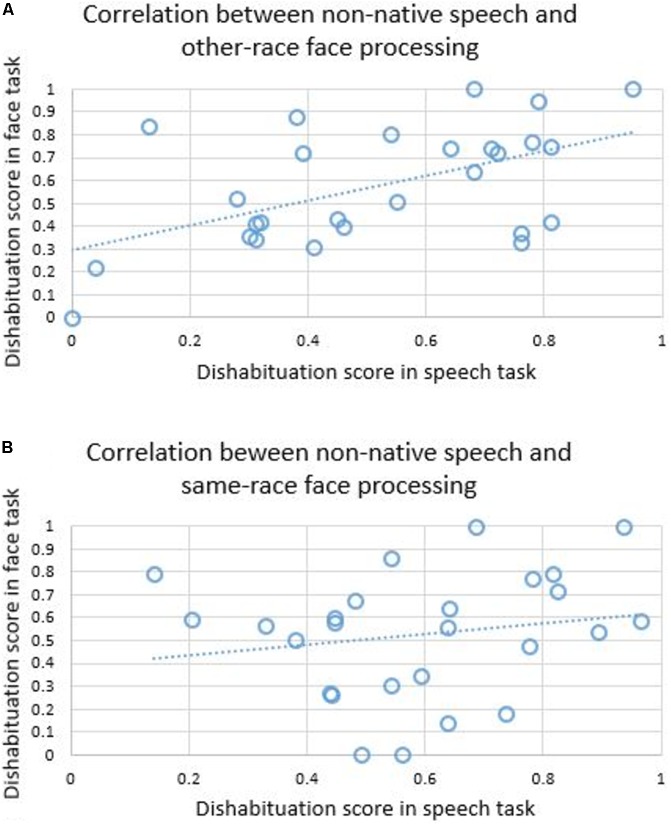
Correlations between dishabituation scores in the non-native speech and other-race face task **(A)**, and between dishabituation scores in the non-native speech and same-race face task **(B)**.

Next, we tested the Pearson correlations (**Figure 2**) between the speech- and face-related dishabituation scores of each infant separately for Group A and Group B. Infants in Group A showed a highly significant correlation between the dishabituation scores in the speech and face tasks [*R*(25) = 0.536, *p* = 0.004], while the infants in Group B did not [*R*(24) = 0.182, *p* = 0.374].

## Discussion

Agreeing with previous research, our results confirmed that 9-month-old monolingual infants were not able to discriminate between non-native tones (Mattock and Burnham, 2006; Yeung et al., 2013; Götz et al., 2018), or between other-race faces to which they had no prior exposure (Kelly et al., 2007, 2009).

Most interestingly, our results showed that the dishabituation scores of infants for non-native tones and other-race faces were highly correlated, while the dishabituation scores for non-native tones and same-race faces showed no correlation. The positive correlation between the ability to discriminate between non-native tones and other-race faces indicates that infants who are weak in discriminating other-race faces are also weak in discriminating non-native speech and vice versa. Most importantly for the interpretation of this effect, no correlation was found between the discrimination of non-native tones and same-race faces, which indicates that the correlation between the dishabituation scores for non-native tones and other-race faces is not merely an effect of general tasks requirements (e.g., attention, memory, or habituation speed). Our results therefore support the hypothesis that the developmental trajectories of perceptual narrowing in speech and faces share some underlying mechanisms that drive these processes and can affect the speed and/or the outcome of these processes across both domains within an individual. It could well be that these domain-general processes are involved in applying statistical learning to the stimuli surrounding infants, allowing for the specialization of their perceptual systems to the stimuli classes which appear most often (Maurer and Werker, 2014). The precise neural organization of these mechanisms would therefore be an important target for future research.

## Author Contributions

AK contributed to the design of the work, acquisition and analysis of the data, and drafting of the manuscript. AG contributed to the design of the work and revising of the manuscript. BH contributed to the design of the work, drafting and revising of the manuscript. GS contributed to the design of the work, analysis of the data, and revising of the manuscript. All authors approved the final version and agreed to be accountable for all aspects of the work in ensuring that questions related to the accuracy or integrity of any part of the work are appropriately investigated and resolved.

## Conflict of Interest Statement

The authors declare that the research was conducted in the absence of any commercial or financial relationships that could be construed as a potential conflict of interest.
